# Is surgery indicated for elderly patients with early stage nonsmall cell lung cancer, in the era of stereotactic body radiotherapy?

**DOI:** 10.1097/MD.0000000000005212

**Published:** 2016-10-28

**Authors:** Nam P. Nguyen, Juan Godinez, Wei Shen, Vincent Vinh-Hung, Helena Gorobets, Juliette Thariat, Fred Ampil, Jacqueline Vock, Ulf Karlsson, Alexander Chi

**Affiliations:** a Department of Radiation Oncology, Howard University, Washington DC; b Department of Radiation Oncology, Rochester General Hospital, Rochester, NY; c Division of Pulmonary Medicine, University of Arizona, Tucson, AZ; d Department of Radiation Oncology, University of Martinique, Martinique, France; e Department of Oral Maxillofacial Surgery, University of Kiev, Kiev, Ukraine; f Department of Radiation Oncology, University of Nice, Nice, France; g Department of Radiation Oncology, Louisiana State University, Shreveport, Louisiana; h Department of Radiation Oncology, Lindenhofspital, Bern, Switzerland; i Department of Radiation Oncology, Marshfield Clinic, Marshfield; j Department of Radiation Oncology, University of West Virginia, Morgantown, West Virginia.

**Keywords:** early stage, elderly, lung cancer, SBRT, surgery

## Abstract

**Background:**

The aim of this article is to assess the influence of comorbidities among elderly patients (at least 70 year old) undergoing surgery for early stage nonsmall cell lung cancer (NSCLC) and to explore the tolerability and efficacy of surgery in relation to stereotactic body radiotherapy (SBRT) in this patient population.

**Methods:**

A review of the literature on the prevalence of comorbidities among elderly patients with early stage NSCLC, and the impact of comorbidity factors on survival following surgery was conducted. Survival rates and the incidence of complications following SBRT for this patient population were also identified.

**Results:**

Comorbidities in elderly patients with early stage NSCLC may preclude surgery or lead to poor survival following surgery. However, chronological age alone should not be used as a deciding factor to deny curative treatment in elderly, but fit patients. Stereotactic body radiotherapy is well tolerated by elderly lung cancer patients and may result in survival rates similar to that following surgery.

**Conclusion:**

SBRT should be the treatment of choice for early stage NSCLC in elderly patients with multiple comorbidities that preclude surgery. The roles of surgery and SBRT for elderly, -fit patients with early stage NSCLC needs to be further defined in future prospective trials.

## 1 Introduction

The standard of care for early stage nonsmall cell lung cancer (NSCLC) is lobectomy due to its high cure rate. However, surgery may lead to a higher peri-operative mortality and poorer survival in elderly lung cancer patients who have a high comorbidity score.^[1]^ Many lung cancer patients are former smokers, thus exposing them to the risk of chronic obstructive pulmonary disease (COPD), coronary artery disease (CAD), and peripheral vascular disease (PVD), which may preclude them from having surgery.^[^2 3^]^ In addition, elderly patients may have other comorbidities, such as hypertension, diabetes, and renal disease, which may increase the complication rates following surgery. This may have accounted for a *decrease* in patient survival.^[^4 5^]^ Recently, stereotactic body radiotherapy (SBRT), a new technique of radiotherapy delivering a high dose of radiation under image guidance, has been introduced for the curative treatment of early stage NSCLC. Preliminary results following SBRT demonstrated high local control rates and minimal complications even among patients whose medical conditions preclude surgery.^[6]^ Among patients who are candidates for surgery but decline lobectomy, a high survival rate has also been reported following SBRT for early stage NSCLC.^[^7 8^]^ Thus, the roles of surgery and SBRT needs to be further defined in elderly patients with early stage NSCLC to identify the most optimal treatment approach in this patient population. In this study, we assess the prevalence of comorbidities in elderly patients with NSCLC, and their impact on the efficacy and tolerability of surgical treatment in these patients. In addition, we review selected studies of surgery and SBRT in elderly patients with early stage NSCLC to further assess the efficacy and tolerance of each treatment in this patient population.

## 2 Materials and methods

A search was conducted based on the PubMed electronic database. The following terms were explored and used for each database search: NSCLC, early stage (stage I), elderly, comorbidity, lobectomy, mortality, survival, and SBRT. A reference list of relevant papers was then searched for additional publications. The following criteria were analyzed in each article: prevalence of comorbidity among elderly lung cancer patients, impact of comorbidity on survival following lobectomy and SBRT, and complications. Articles describing the survival of elderly cancer patients (70 year old or older) following surgery or SBRT were selected. The age cutoff of 70 was chosen because of anthropometric studies reporting increased weight loss and sarcopenia in men and women after the age of 70.^[^9 10^]^ Sarcopenia has been linked to increased risk of complications and poorer survival after oncologic surgery.^[11]^ The comorbidity index was identified in each article. Only studies reported in English were included. Duplicate articles were excluded.

The University of Arizona Institutional Review Board approved the study as it is a review of the literature and does not require patient consent.

## 3 Results

**Figure 1:**
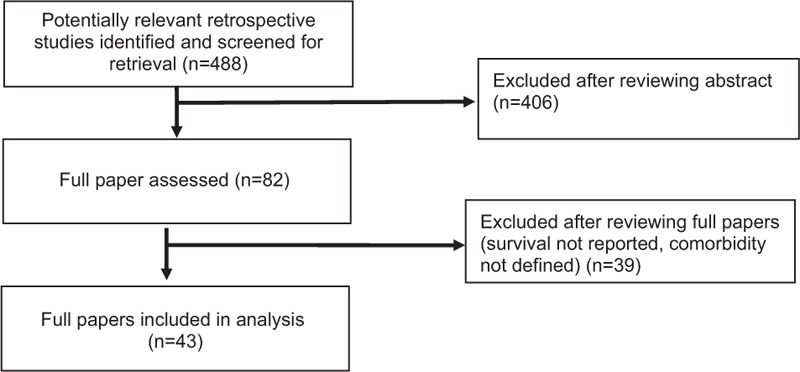
Prisma flow diagram of included studies.

A total of 488 articles were screened. However, 82 articles were reviewed. Also, 39 studies were excluded as they did not fit selection criteria. Figure 1 summarizes the Prisma flow diagram. The Charlson comorbidity index was the most common index to analyze complications and mortality. Eighteen studies linked the prevalence of comorbidity and age among lung cancer patients. However, 29 studies and 6 studies reported survival and complications rates among patients who were at least 70 year old who underwent surgery or SBRT for early stage NSCLC, respectively. All studies were retrospective. Thus, selection bias cannot be avoided. Patient selection criteria were identified in each study and reported in the results section.

### 3.1 Prevalence of comorbidity among elderly lung cancer patients

The severity of the comorbidity score increased with age. Among patients who underwent chemotherapy and radiotherapy for lung cancer, Charlson scores of >2 was only 8% for those <60 year old but increased to 26% and 43% for patients between 60- and 69 year old and 70- and 79-year old, respectively.^[12]^ Other studies also showed an increase of prevalence and severity of comorbidity among elderly cancer patients using different indexes.^[^13–21^]^ The severity of comorbidity scores may be related to the number of comorbidities in the elderly: 44.7% of patients >80 years had >3 comorbidities.^[18]^

Among studies that used the Charlson comorbidity index, the comorbidity score of grade 2 or higher ranged from 34.3% to 68.5% for patients >80.^[^12 18 21^]^ Table 1 summarizes the prevalence and severity of comorbidity according to age in patients with NSCLC.

**Table 1:**
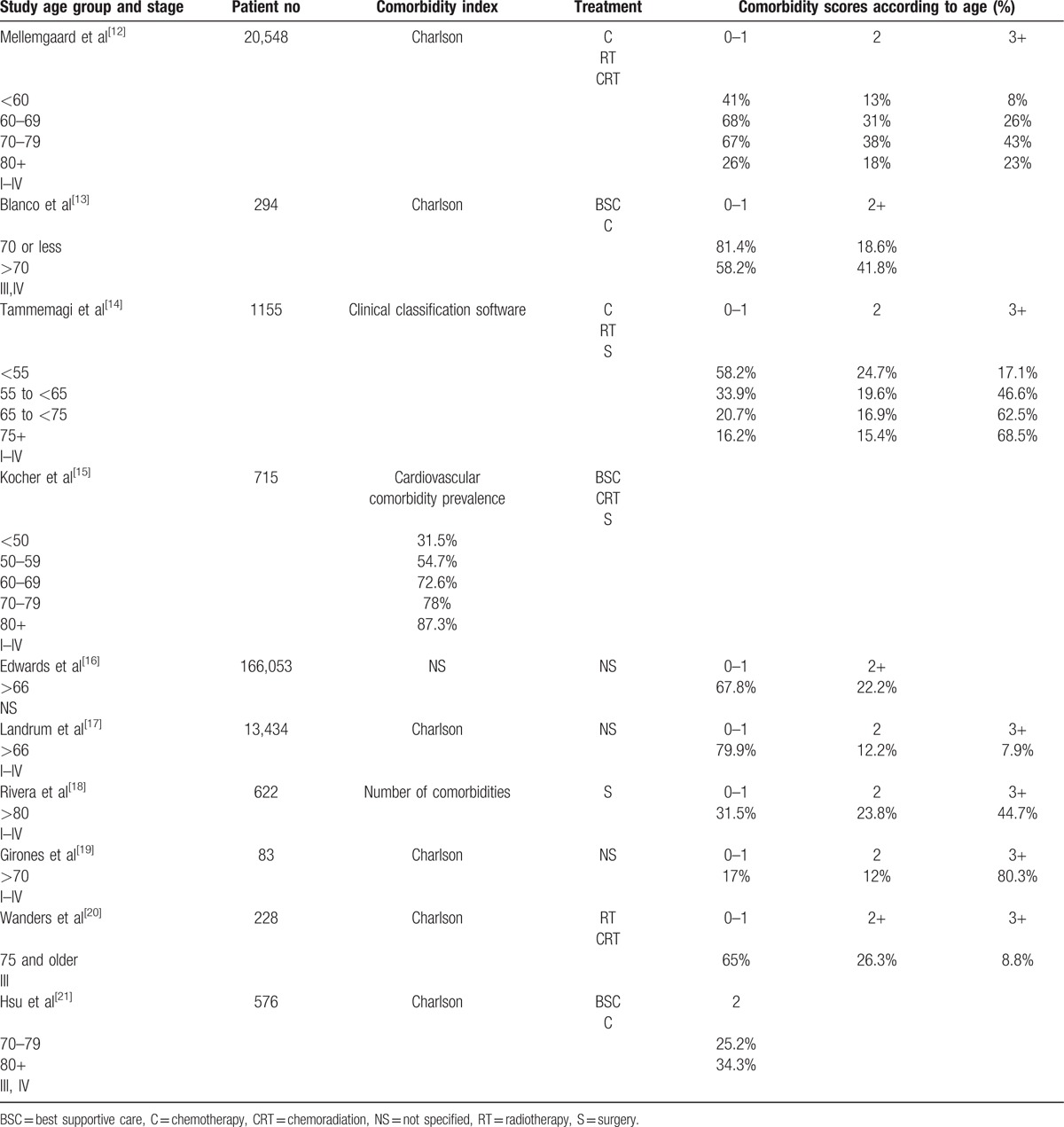
Prevalence and severity of comorbidity according to age in patients with nonsmall cell lung cancer.

### 3.2 Morbidity and mortality of surgery in elderly patients with early stage nonsmall cell lung cancer

Surgery with either lobectomy or pneumonectomy was performed in elderly lung cancer patients 70, 75, or 80 year old or older.^[^22–51^]^ Patients selected for surgery were often physically fit and underwent extensive cardio-pulmonary evaluation prior to surgery. Only 5 studies correlated comorbidity index with complications or survival.^[^22 23 35 41 42^]^ Among studies which included patients who were at least 70 year old, the mortality, and complications rates ranged from 1.1% to 22.2% and 7.4% to 55.2%, respectively.^[^22–36^]^ Survival of those patients ranged from 20% to 57% at 5 years. In the 2 studies reporting on patients who were at least 75 year old, the mortality, complications, and overall survival rates were 2.3% and 2.7%, 18.6% and 60%, and 65% and 68% at 3 and 5 years, respectively.^[^37 38^]^ In patients who were at least 80 year old, these rates at 5 years ranged from 1.2% to 12%, 8.4% to 50%, and 19.1% and 65.9% respectively.^[^39–50^]^ Complications rates increased whereas survival decreased for patients with increased comorbidity scores.^[^22 23 35 41 42^]^ Table 2 summarizes the morbidity and mortality of elderly lung patients who underwent surgery for early stage NSCLC.

**Table 2:**
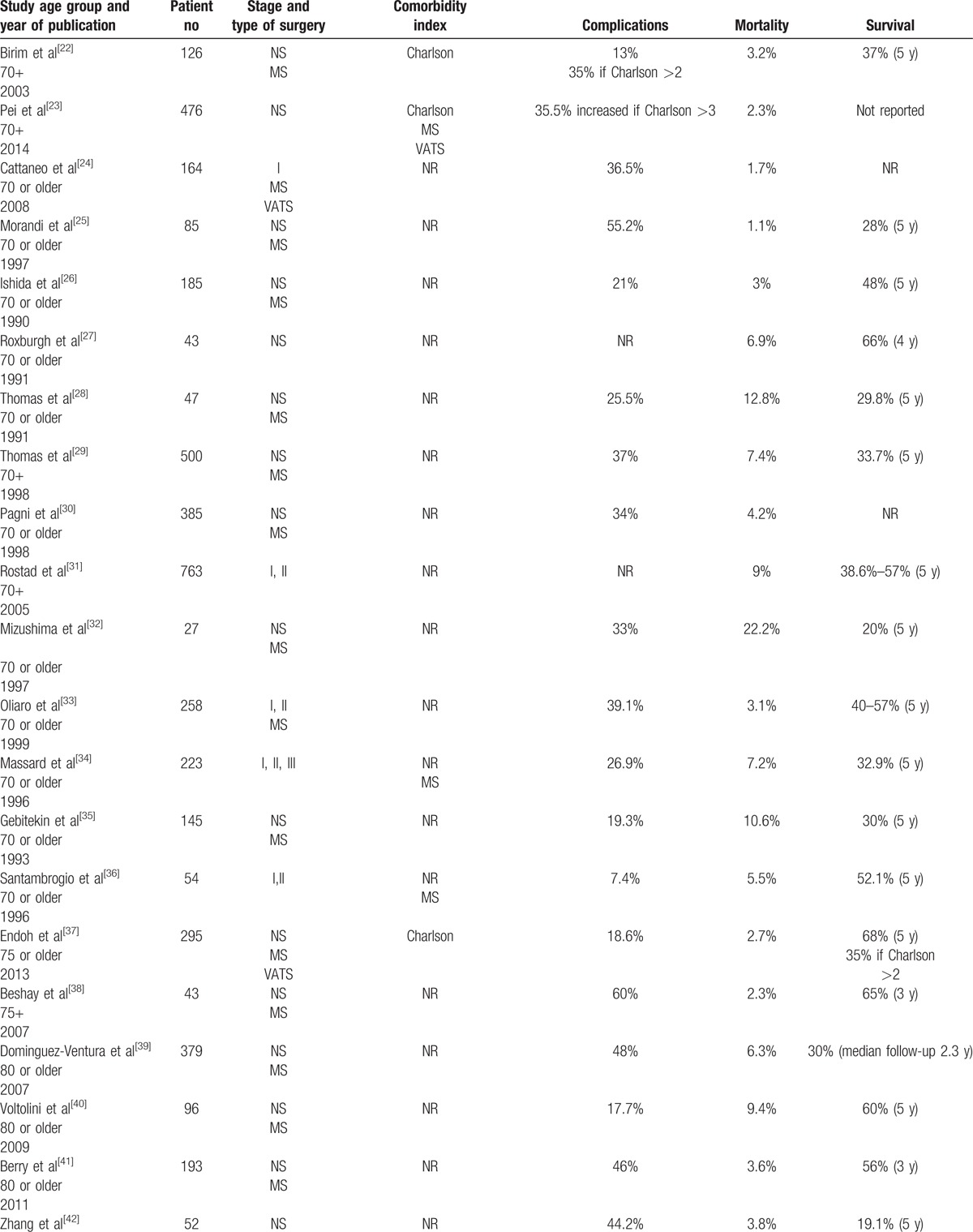
Morbidity and mortality of surgery in elderly patients with early stages nonsmall cell lung cancer.

**Table 2 (Continued):**
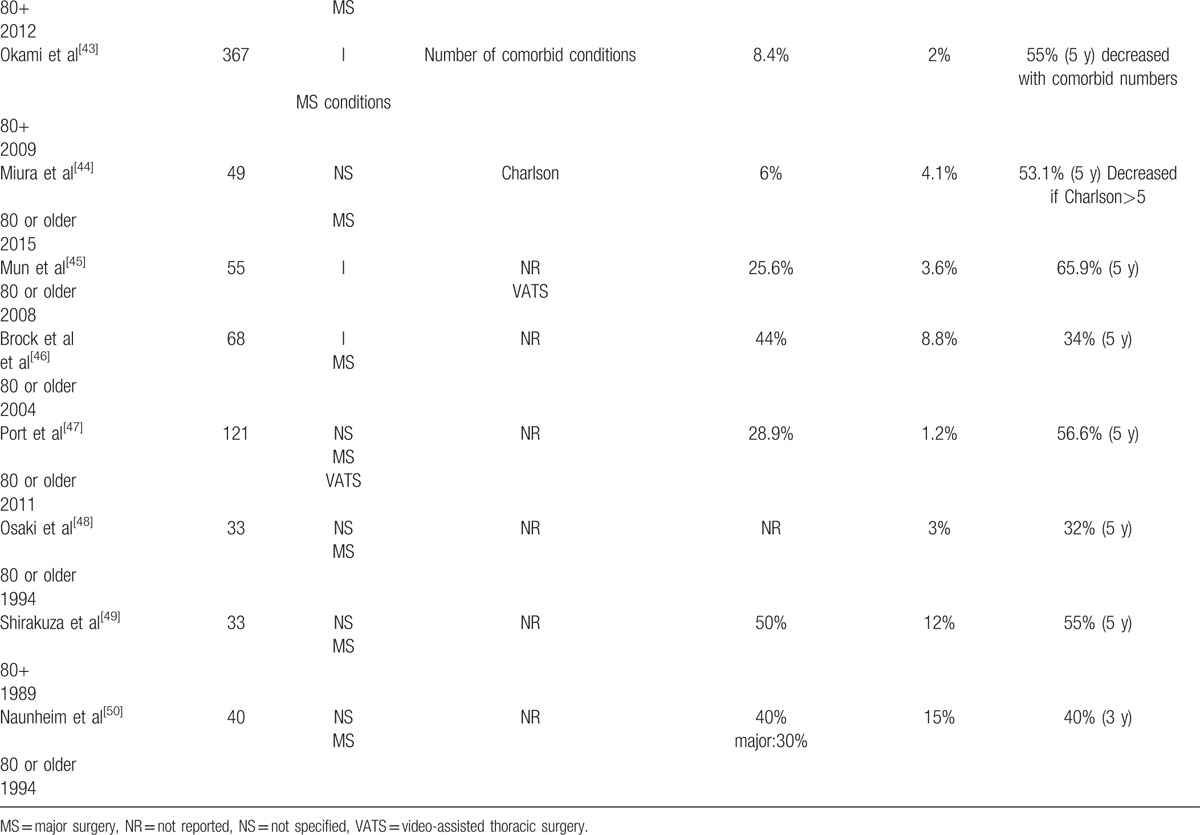
Morbidity and mortality of surgery in elderly patients with early stages nonsmall cell lung cancer.

### 3.3 Morbidity and mortality of stereotactic body radiotherapy in elderly patients with early stage nonsmall cell lung cancer

Patients selected for SBRT were often excluded from surgery because of existing comorbidities. Mortality and complication rates of elderly patients with early stage NSCLC who underwent SBRT ranged from 0% to 1.7%, and 0.9% to 10% respectively.^[^52–57^]^ The 3-year survival ranged from 40.7% to 53%. Three studies reported the Charlson comorbidity scores but did not correlate the index severity with survival or complications. Table 3 summarizes the morbidity and mortality of elderly patients who underwent SBRT for early stage NSCLC.

**Table 3:**
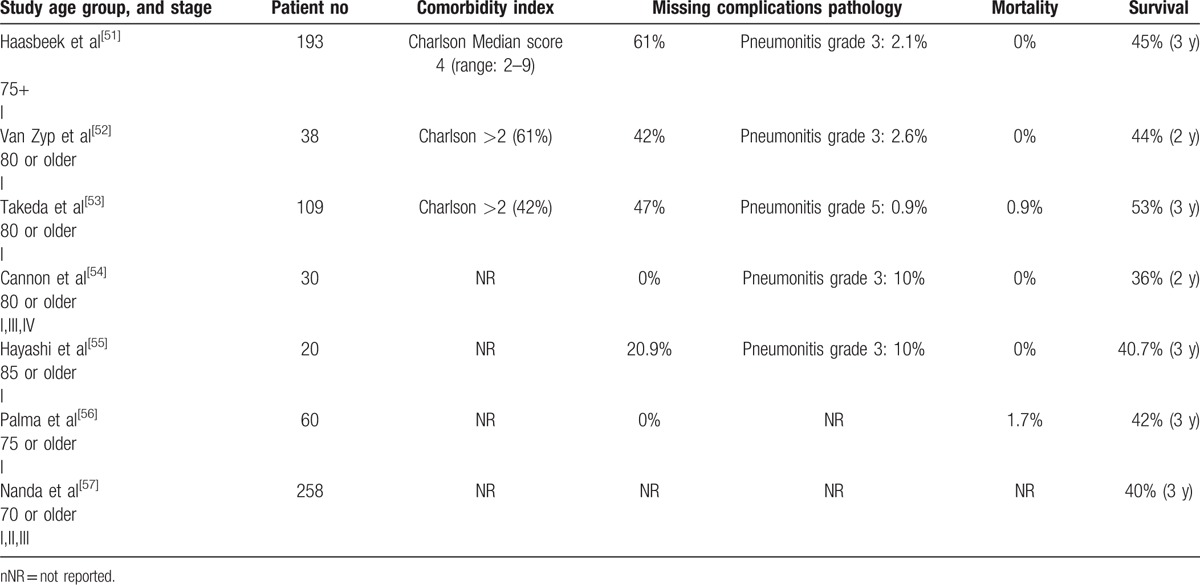
Morbidity and mortality of stereotactic body radiotherapy in elderly patients with early stage nonsmall cell lung cancer.

## 4 Discussion

Treatment of elderly patients with early stage NSCLC remains a challenge because of the presence of comorbidity factors. The Lung Cancer Study Group identified increasing age as a risk factor for mortality in patients undergoing surgery for NSCLC. Mortality rates were 1.3%, 4.1%, and 7.1% for patients <60 year old, 60 to 69 year old, and 70 year old or older, respectively.^[58]^ Postoperative death occurred in 25% of patients aged 80 or older compared to 5% of those <80.^[59]^ Compared to other cancers such as breast and colorectal cancers, the mortality and complication rates following surgery for NSCLC are higher because of reduced cardio-pulmonary reserve.^[59]^ Even though it is still controversial, the Charlson comorbidity index has been frequently used to assess the influence of comorbidity factors on survival following surgery for NSCLC.^[^60–62^]^ As an illustration, among patients of all ages undergoing surgery for NSCLC, a 5-year survival of 52%, 48%, and 28% has been reported for patients with Charlson comorbidity grade 0, 1 to 2, and >2 respectively.^[60]^ The poor survival associated with a high Charlson score for NSCLC has been shown in other prospective studies.^[^61 62^]^ Thus, patients who are selected for surgery usually are in excellent physical condition, with adequate cardiopulmonary function, and had extensive *peri-operative* evaluation.^[^34 39 42^]^ In addition, special surgical procedures such as video-assisted thoracoscopic surgery (VATS) may further reduce the incidence of surgical complications, thus allowing surgery in octogenarians.^[47]^ Consequently, 5-year survival rates between 60% and 65.9% has been observed in selected studies for patients 80 year old or older.^[^40 45^]^ Thus, chronological age should not be a contraindication to curative surgery in all elderly patients with early stage NSCLC. Instead, priority should be given to the evaluation of comorbidities and performance status when evaluating these patients for surgery. Higher complications rates and poorer survival were reported among patients with high comorbidity scores in selected studies that take into consideration the influence of comorbidity on surgical outcomes.^[^22 23 43 44 50^]^ For example, Endoh et al^[37]^ reported a 5-year survival of 68% for patients who were at least 75 year old. However, the 5-year survival was reduced to 35% among patients with a Charlson score above 2 even though VATS was one of the surgical techniques being reported on this study. The impact of medical comorbidities on the survival of elderly lung cancer patients was also corroborated in other studies.^[^43 44^]^ Existing evidence suggests that elderly NSCLC patients with severe comorbidity scores may not be the best candidates for surgery. Given the fact that the number of comorbidities increases with age, a significant portion of elderly NSCLC patients with severe comorbidity score would be precluded from surgery.^[^18 19^]^ Those patients would likely benefit from SBRT as an alternative for cure.^[6]^ Among elderly NSCLC patients who underwent SBRT, 3-year overall survival ranged from 40.7% to 53% despite a high Charlson score.^[^51 52 55^]^ Excellent local control between 83% to 100% was observed with minimal morbidity.^[^52–53^]^ Mortality rates were very low (0–1.7%). The most serious complication was grade 3 radiation-related pneumonitis which was reversible and ranged from 2.1% to 10%. Even though the duration of follow-up in SBRT studies (only 3-year survival to date) is relatively short when compared with that in surgical series, current evidence suggests that SBRT should be the treatment of choice for elderly patients with early stage NSCLC in whom medical conditions preclude surgery.

The rate of serious complications is still significant (28.9%) even in physically fit elderly NSCLC patients with minimal comorbidities receiving a less morbid surgical procedure such as VATS.^[44]^ Even though there is still controversy, VATS has been associated with fewer complications and shortened hospital stay when compared with open thoracotomy.^[^63 64^]^ Surgical complications were mostly cardiopulmonary and ranged from 8.4% to 60% (Table 2 ). These complications may affect patients’ quality of life because of the longer recovery time when compared with younger patients.^[65]^ As the mortality and complications rates of SBRT are significantly less when compared with that following VATS for early stages NSCLC, elderly fit patients may benefit from SBRT if survival comparable to that following surgery can be achieved.^[6]^ A pooled randomized study reported a 3-year survival of 95% and 79% for SBRT and surgery respectively.^[7]^ In this study, grade 3 complications were 10% and 44% for SBRT and surgery, respectively. There was no death in the SBRT arm. One patient in the surgical arm died from complications. Even though the number of patients in this study is small, the study highlights that while both treatment modalities are effective for early stage NSCLC, less complications were observed following SBRT. A matched-pair comparison of SBRT versus surgery for stage I NSCLC also corroborated similar survival rates between the 2 treatments.^[66]^ These studies raise the intriguing questions of whether SBRT may become the treatment of choice for elderly lung cancer patients with early stage NSCLC lung cancer regardless of the presence of comorbidity factors, or physical status. Another argument for SBRT is that it costs less when compared with lobectomy. Among 486 patients at least 66 year old who underwent surgery or SBRT, the estimated Medicare cost over 5 years was 54,968 and 82,641 dollars for SBRT and lobectomy, respectively.^[67]^ As the number of elderly lung cancer patients is rapidly rising in the western world, prospective randomized studies should be conducted in the future to compare the cost-effectiveness of SBRT and surgery for this age group.^[68]^ The effectiveness of SBRT to improve local control and survival while minimizing treatment complications for lung cancer may be related to its ability to precisely target the cancer with an ablative dose of radiation while sparing the normal thoracic organs such as the heart and the lung. Daily pre-treatment tumor localization under image guidance allows the radiation oncologist to reduce the amount of normal lung tissue surrounding the tumor to be included in the PTV (planning target volume to avoid marginal miss), thus minimizing the volume of normal lung tissue to be irradiated to a high radiation dose. In addition, special devices to reduce respiration-induced tumor movement, such as the active breathing control or the abdominal compression device, further decrease excessive lung irradiation which can be substantial if the tumor is located in lower lobe. As a result, there was little change in pulmonary function following SBRT for lung cancer.^[69]^ In another study of 55 medically inoperable patients who underwent SBRT for early stage lung cancer and who had regular pulmonary function test studies (PFTs) every 3 months for the first 2 years and every 6 months after treatment, the mean percentage of forced expiratory volume in the first second (FEV1) and diffusing capacity for carbon monoxide declined by 5.8% and 6.3%, respectively.^[70]^ There were also no significant changes in arterial blood gas and oxygen saturation after 2 years.^[70]^ These results corroborated with the low morbidity observed following SBRT among patients who required chronic oxygen therapy because of lung damage prior to radiotherapy^[71]^ and highlighted the safety of SBRT for elderly lung cancer patients regardless of baseline lung function.^[72]^ Follow-up of a pooled randomized study comparing surgery to SBRT in patients with operable early stage NSCLC suggested that patients’ global heath quality of life may be better with SBRT because of the low morbidity observed but these results should be confirmed in future prospective studies.^[73]^

We acknowledge the limitations of the current study. In some of the studies reported outside the USA, the absence of pathologic confirmation of cancer in a significant number of patients may raise the question of whether those lung lesions may be benign.^[^52 53^]^ However, excellent local control with minimal complications has been reported following SBRT for locally more advanced stage (II) NSCLC (>5 cm) in US studies where pathological diagnosis of cancer is practically a legal requirement because of *potential* lawsuit for misdiagnosis, which highlights the safety of this new radiotherapy technique.^[74]^ Another limitation of SBRT for early stage NSCLC as a treatment approach is the lack of pathologic mediastinal lymph node information which precludes adjuvant systemic treatment which may impair patient survival when compared with surgery. Unless further randomized trials are performed to compare these 2 treatment modalities, these questions will remain unanswered. We recognize the difficulty to conduct randomized trials among elderly cancer patients because of the bias including patient preference, physicians’ reluctance to treat patient in a curative modality because of their age, and the absence of consensus in treating elderly cancer patients. However, such studies will be necessary in the future to guide the treatment of a growing number of elderly cancer patients across the world,^[75]^ which will require collaborations among the nations and the physicians of various specialties.

## 5 Conclusions

Elderly cancer patients with early stage NSCLC should undergo curative treatment with either surgery or SBRT. Patients with serious comorbidity precluding surgery should have SBRT. The role of surgery and SBRT for the management of elderly fit patients should be tested in future randomized studies.

## Acknowledgments

The authors thank Dayleen De Riggs and Kristen Young for their help in the preparation of the manuscript.
